# PCR Primers for Metazoan Nuclear 18S and 28S Ribosomal DNA Sequences

**DOI:** 10.1371/journal.pone.0046180

**Published:** 2012-09-25

**Authors:** Ryuji J. Machida, Nancy Knowlton

**Affiliations:** 1 Biodiversity Research Center, Academia Sinica, Taipei, Taiwan; 2 National Museum of Natural History, Smithsonian Institution, Washington, D.C., United States of America; Argonne National Laboratory, United States of America

## Abstract

**Background:**

Metagenetic analyses, which amplify and sequence target marker DNA regions from environmental samples, are increasingly employed to assess the biodiversity of communities of small organisms. Using this approach, our understanding of microbial diversity has expanded greatly. In contrast, only a few studies using this approach to characterize metazoan diversity have been reported, despite the fact that many metazoan species are small and difficult to identify or are undescribed. One of the reasons for this discrepancy is the availability of universal primers for the target taxa. In microbial studies, analysis of the 16S ribosomal DNA is standard. In contrast, the best gene for metazoan metagenetics is less clear. In the present study, we have designed primers that amplify the nuclear 18S and 28S ribosomal DNA sequences of most metazoan species with the goal of providing effective approaches for metagenetic analyses of metazoan diversity in environmental samples, with a particular emphasis on marine biodiversity.

**Methodology/Principal Findings:**

Conserved regions suitable for designing PCR primers were identified using 14,503 and 1,072 metazoan sequences of the nuclear 18S and 28S rDNA regions, respectively. The sequence similarity of both these newly designed and the previously reported primers to the target regions of these primers were compared for each phylum to determine the expected amplification efficacy. The nucleotide diversity of the flanking regions of the primers was also estimated for genera or higher taxonomic groups of 11 phyla to determine the variable regions within the genes.

**Conclusions/Significance:**

The identified nuclear ribosomal DNA primers (five primer pairs for 18S and eleven for 28S) and the results of the nucleotide diversity analyses provide options for primer combinations for metazoan metagenetic analyses. Additionally, advantages and disadvantages of not only the 18S and 28S ribosomal DNA, but also other marker regions as targets for metazoan metagenetic analyses, are discussed.

## Introduction

Human activities pose severe threats to planetary biodiversity e.g. [1]–[3], and it is thus critically important to be able to rapidly estimate biodiversity across space and through time. Species richness is the most widely used index of diversity, but it is difficult to estimate diversity comprehensively using traditional morphological approaches because for many groups most species remain undescribed [4]–[6], and even when described, are often difficult to identify. In the marine environment, species that comprise the majority of metazoan biodiversity are also often small and thus difficult to sample and analyze individually (for example Nematoda, Copepoda, Ostracoda, Rotifera, Kinorhyncha, Loricifera, and Tardigrada). In this context, the ability to rapidly assess biodiversity at various spatio-temporal scales without assigning formal taxonomic names to all samples is urgently needed.

Analyses based on second-generation sequencing have many advantages in this regard, as they can produce very large numbers of sequences from single samples, either by targeting single or multiple genes using PCR (metagenetics: [7]) or by targeting entire genomes (metagenomics). However, most metagenetic studies to date have focused on microbes and protozoans e.g. [8]–[11], and only a limited number of studies have been carried out for metazoans [12]–[17].

Unlike the situations with microbes, where analysis of the 16S ribosomal DNA sequence is standard [18], the appropriate gene for metazoan metagenetic studies is less clear [13]. Over the past 20 years, several studies have reported universal primers for metazoan nuclear 18S and 28S ribosomal DNA sequences [19]–[21], but the extent of compatibility between the primers and the target regions of these primers has not been estimated thoroughly. In 1994, Hillis and Dixon [19] reported universal primers for nuclear ribosomal DNA regions using up to seven reference sequences. Since then, much larger numbers of reference sequences have been made available in databases, and the numbers are expected to continue to increase.

In the present study, we aimed to perform (i) discovery of new metazoan universal primers, (ii) estimation of compatibility of the newly designed and also previously reported primers, and (iii) identification of regions with higher variability within the genes. By integrating results obtained from these analyses, we propose combinations of primers that are likely to retrieve a more complete representation of the taxonomic diversity of metazoans present in environmental samples.

Our primers might be applicable not only for environmental samples but also for individual samples. However, the goal of this study was to design primers that amplify a short portion of target genes for metagenetic analyses, rather than for phylogenetic analyses for which longer sequences of these genes would be preferred.

## Results and Discussion

**Figure 1:**
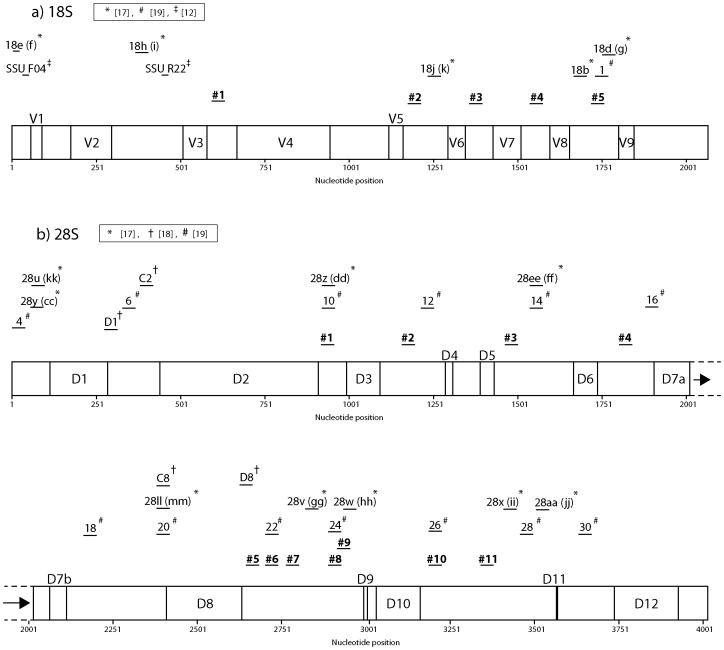
Relative positions of the newly designed primers and primers identified from previous studies [19]–[21] depicted on linear maps of 18S (a) and 28S (b) ribosomal DNA. Primers analyzed in this study are depicted in bold. Primers #6, #8, and #10 designed on 28S (b) ribosomal DNA are modified from 22, 24, 26 of [21], respectively. Primers used in Fonseca et al. [12] were also drawn. Relative positions of variable region (V) and expansion segments (D) [22] based on the secondary structure model of Apis mellifera ribosomal RNA genes are also depicted.

We identified five and eight conserved regions that were suitable for designing PCR primers against nuclear 18S and 28S ribosomal DNA (Table 1). In addition to the newly designed primers, we also tested the efficacy of previously published primers by comparing sequence similarity between the primers and SILVA datasets [19]–[21] (see materials and methods, Fig. 1, Tables S1, S2). From these, we identified three additional primers for the 28S ribosomal DNA region (primer numbers 22, 24, and 26 of [21]) that were also used in the compatibility test described below.

**Table 1:** List of the 18S and 28S ribosomal DNA primers designed in this study.

18S ribosomal DNA		
Forward primers (3′ to 5′)	Reverse complement primers (3′ to 5′)	Degeneracy
#1: CTGGTGCCAGCAGCCGCGGYAA		2
#2: AACTTAAAGRAATTGACGGA	#2_RC: TCCGTCAATTYCTTTAAGTT	2
#3: GYGGTGCATGGCCGTTSKTRGTT	#3_RC: AACYAMSAACGGCCATGCACCRC	16
#4: ATAACAGGTCWGTRATGCCCTYMG	#4_RC: CKRAGGGCATYACWGACCTGTTAT	16
	#5_RC: GTGTGYACAAAGGBCAGGGAC	6
28S ribosomal DNA		
Forward primers (3′ to 5′)	Reverse complement primers (3′ to 5′)	Degeneracy
#1: CCGTCTTGAAACACGGDCYRAG		6
#2: AGGGGCGAAAGACYAATCGAA	#2_RC: TTCGATTRGTCTTTCGCCCCT	2
#3: TTTTGGTAAGCAGAACTGGYG	#3_RC: CRCCAGTTCTGCTTACCAAAA	2
#4: GATCTYRGTGGYAGTAGCRAVT	#4_RC: ABTYGCTACTRCCACYRAGATC	48
#5: GGGAATCYRACTGTHTAATTAAA	#5_RC: TTTAATTADACAGTYRGATTCCC	12
#6: TGATTTCTGCCCAGTGCTYWGAAWGT	#6_RC: ACWTTCWRAGCACTGGGCAGAAATCA	8
#7: AACGGCGGRRGTAACTATGACTYT	#7_RC: ARAGTCATAGTTACYYCCGCCGTT	8
#8: GGGAAAGAAGACCCTGTTGAG	#8_RC: CTCAACAGGGTCTTCTTTCCC	1
#9: AAGACCCTGTTGAGYTTGACTCT	#9_RC: AGAGTCAARCTCAACAGGGTCTT	2
#10: GGGAGTTTGRCTGGGGCGG	#10_RC: CCGCCCCAGYCAAACTCCC	2
	#11_RC: GCTTGGCBGCCACAAGCCAGTTA	3

Primers 28S #6, 28S #8, and 28S #10 were modified from primers 22, 24, 26 [21], respectively. Primers ultimately selected for best overall efficacy are highlighted in bold.

### 1. Compatibility test between the primers and SILVA datasets

To test the potential efficacy of primers, the similarity between primer sequences and target regions from the SILVA database sequences were compared for each phylum (Tables 2, 3). These sequences represent the 18S and 28S ribosomal DNA sequences for 34 and 27 phyla, respectively. However, only 24 of 34 phyla for the 18S ribosomal DNA (Platyhelminthes, Chaetognatha, Chordata, Echinodermata, Hemichordata, Annelida, Brachiopoda, Bryozoa, Entoprocta, Mollusca, Myzostomida, Nemertea, Arthropoda, Tardigrada, Sipuncula, Acanthocephala, Cycliophora, Gastrotricha, Nematoda, Nematomorpha, Rotifera, Cnidaria, Ctenophora, and Porifera) and 11 of 27 phyla for the 28S ribosomal DNA (Platyhelminthes, Chordata, Annelida, Bryozoa, Mollusca, Arthropoda, Acanthocephala, Nematoda, Rotifera, Cnidaria, and Porifera) had 10 or more sequences in the database at the time of the analysis. The remaining phyla typically had five or fewer sequences in the database, and the generality of our findings for these groups is thus limited.

**Table 2:** Percentages of sequences, which showed mismatches between the primer and target regions of the nuclear 18S ribosomal DNA sequences downloaded from the SILVA database.

Phylum (# sequences)										
	Primer # 1		Primer # 2		Primer # 3		Primer # 4		Primer # 5	
	% (# Sequences)		% (# Sequences)		% (# Sequences)		% (# Sequences)		% (# Sequences)	
	One	Two or more	One	Two or more	One	Two or more	One	Two or more	One	Two or more
Total										
Metazoa										
Eumetazoa										
Bilateria (18)	11.11 (2)	5.56 (1)	16.67 (3)	0.00 (0)	0.00 (0)	0.00 (0)	0.00 (0)	0.00 (0)	0.00 (0)	0.00 (0)
Acoelomata										
Platyhelminthes (1003)	1.20 (12)	1.69 (17)	1.50 (15)	0.50 (5)	1.79 (18)	0.80 (8)	5.08 (51)	0.80 (8)	1.10 (11)	0.50 (5)
Coelomata										
Deuterostomia										
Chaetognatha (20)	0.00 (0)	5.00 (1)	5.00 (1)	15.00 (3)	10.00 (2)	0.00 (0)	25.00 (5)	0.00 (0)	0.00 (0)	5.00 (1)
Chordata (763)	6.03 (46)	7.08 (54)	3.28 (25)	8.39 (64)	3.15 (24)	10.75 (82)	6.55 (50)	1.44 (11)	6.82 (52)	4.59 (35)
Echinodermata (156)	1.28 (2)	2.56 (4)	1.92 (3)	0.64 (1)	1.92 (3)	0.00 (0)	1.28 (2)	0.00 (0)	0.64 (1)	1.28 (2)
Hemichordata (23)	0.00 (0)	4.35 (1)	0.00 (0)	0.00 (0)	0.00 (0)	4.35 (1)	0.00 (0)	0.00 (0)	0.00 (0)	0.00 (0)
Xenoturbellida (1)	0.00 (0)	0.00 (0)	0.00 (0)	0.00 (0)	0.00 (0)	0.00 (0)	0.00 (0)	0.00 (0)	0.00 (0)	0.00 (0)
Protostomia										
Annelida (983)	0.51 (5)	1.32 (13)	0.71 (7)	1.02 (10)	1.12 (11)	1.32 (13)	1.53 (15)	0.71 (7)	1.53 (15)	0.92 (9)
Echiura (4)	0.00 (0)	0.00 (0)	25.00 (1)	0.00 (0)	0.00 (0)	0.00 (0)	0.00 (0)	0.00 (0)	0.00 (0)	0.00 (0)
Brachiopoda (41)	0.00 (0)	2.44 (1)	2.44 (1)	0.00 (0)	0.00 (0)	4.88 (2)	2.44 (1)	0.00 (0)	0.00 (0)	0.00 (0)
Bryozoa (47)	0.00 (0)	0.00 (0)	0.00 (0)	2.13 (1)	0.00 (0)	2.13 (1)	2.13 (1)	2.13 (1)	2.13 (1)	0.00 (0)
Entoprocta (14)	0.00 (0)	7.14 (1)	7.14 (1)	0.00 (0)	0.00 (0)	0.00 (0)	0.00 (0)	0.00 (0)	0.00 (0)	0.00 (0)
Mollusca (887)	0.68 (6)	0.45 (4)	0.68 (6)	2.37 (21)	1.24 (11)	1.69 (15)	0.56 (5)	0.79 (7)	0.45 (4)	0.90 (8)
Myzostomida (36)	2.78 (1)	0.00 (0)	2.78 (1)	0.00 (0)	0.00 (0)	0.00 (0)	0.00 (0)	0.00 (0)	0.00 (0)	0.00 (0)
Nemertea (34)	0.00 (0)	0.00 (0)	2.94 (1)	0.00 (0)	0.00 (0)	0.00 (0)	0.00 (0)	0.00 (0)	0.00 (0)	0.00 (0)
Panarthropoda										
Arthropoda (7591)	5.03 (382)	1.21 (92)	1.49 (113)	0.84 (64)	2.69 (204)	0.91 (69)	3.90 (296)	1.26 (96)	3.19 (242)	0.69 (52)
Onychophora (2)	0.00 (0)	0.00 (0)	0.00 (0)	0.00 (0)	0.00 (0)	0.00 (0)	0.00 (0)	0.00 (0)	0.00 (0)	0.00 (0)
Tardigrada (102)	2.94 (3)	0.98 (1)	0.00 (0)	0.00 (0)	4.90 (5)	0.00 (0)	1.96 (2)	0.00 (0)	0.00 (0)	0.00 (0)
Priapulida (6)	0.00 (0)	0.00 (0)	0.00 (0)	0.00 (0)	0.00 (0)	0.00 (0)	0.00 (0)	0.00 (0)	0.00 (0)	0.00 (0)
Sipuncula (16)	0.00 (0)	0.00 (0)	6.25 (1)	0.00 (0)	0.00 (0)	0.00 (0)	0.00 (0)	0.00 (0)	0.00 (0)	0.00 (0)
Pseudocoelomata										
Acanthocephala (46)	0.00 (0)	2.17 (1)	2.17 (1)	0.00 (0)	15.22 (7)	0.00 (0)	2.17 (1)	0.00 (0)	6.52 (3)	2.17 (1)
Cycliophora (18)	0.00 (0)	0.00 (0)	0.00 (0)	0.00 (0)	0.00 (0)	0.00 (0)	0.00 (0)	0.00 (0)	0.00 (0)	0.00 (0)
Gastrotricha (17)	0.00 (0)	5.88 (1)	0.00 (0)	0.00 (0)	5.88 (1)	11.76 (2)	5.88 (1)	29.41 (5)	11.76 (2)	5.88 (1)
Kinorhyncha (6)	16.67 (1)	0.00 (0)	0.00 (0)	0.00 (0)	0.00 (0)	0.00 (0)	0.00 (0)	0.00 (0)	0.00 (0)	0.00 (0)
Loricifera (1)	0.00 (0)	0.00 (0)	0.00 (0)	0.00 (0)	0.00 (0)	0.00 (0)	0.00 (0)	0.00 (0)	0.00 (0)	0.00 (0)
Micrognathozoa (2)	0.00 (0)	0.00 (0)	0.00 (0)	0.00 (0)	0.00 (0)	0.00 (0)	0.00 (0)	0.00 (0)	0.00 (0)	0.00 (0)
Nematoda (1375)	2.04 (28)	1.67 (23)	3.05 (42)	0.36 (5)	1.53 (21)	0.87 (12)	1.67 (23)	1.60 (22)	7.05 (97)	1.96 (27)
Nematomorpha (11)	0.00 (0)	9.09 (1)	0.00 (0)	0.00 (0)	0.00 (0)	0.00 (0)	0.00 (0)	9.09 (1)	0.00 (0)	0.00 (0)
Rotifera (68)	1.47 (1)	8.82 (6)	10.29 (7)	0.00 (0)	7.35 (5)	0.00 (0)	0.00 (0)	1.47 (1)	10.29 (7)	0.00 (0)
Cnidaria (969)	4.75 (46)	1.44 (14)	12.28 (119)	4.85 (47)	1.75 (17)	1.24 (12)	3.20 (31)	1.03 (10)	13.31 (129)	8.88 (86)
Ctenophora (18)	0.00 (0)	5.56 (1)	0.00 (0)	0.00 (0)	0.00 (0)	0.00 (0)	0.00 (0)	0.00 (0)	0.00 (0)	0.00 (0)
Mesozoa (5)	0.00 (0)	40.00 (2)	0.00 (0)	20.00 (1)	0.00 (0)	0.00 (0)	0.00 (0)	80.00 (4)	0.00 (0)	0.00 (0)
Placozoa (9)	0.00 (0)	22.22 (2)	0.00 (0)	0.00 (0)	0.00 (0)	0.00 (0)	0.00 (0)	0.00 (0)	0.00 (0)	0.00 (0)
Porifera (211)	1.42 (3)	1.90 (4)	1.42 (3)	0.95 (2)	0.95 (2)	0.00 (0)	0.47 (1)	0.95 (2)	0.00 (0)	1.90 (4)

Comparisons were made for each phylum. One and two or more mismatches were estimated independently. The numbers in parentheses indicate the number of sequences that had the mismatches. The hierarchy of the NCBI taxonomy database is followed in this table.

**Table 3:** Percentages of sequences, which showed mismatches between the primer and target region of the nuclear 28S ribosomal DNA sequences downloaded from the SILVA database.

Phylum (# sequences)																						
	Primer # 1		Primer # 2		Primer # 3		Primer # 4		Primer # 5		Primer # 6		Primer # 7		Primer # 8		Primer # 9		Primer # 10		Primer # 11	
	% (# Sequences)		% (# Sequences)		% (# Sequences)		% (# Sequences)		% (# Sequences)		% (# Sequences)		% (# Sequences)		% (# Sequences)		% (# Sequences)		% (# Sequences)		% (# Sequences)	
	One	Two or more	One	Two or more	One	Two or more	One	Two or more	One	Two or more	One	Two or more	One	Two or more	One	Two or more	One	Two or more	One	Two or more	One	Two or more
Total																						
Metazoa																						
Eumetazoa																						
Acoelomata																						
Platyhelminthes (127)	0.00 (0)	2.36 (3)	7.09 (9)	0.00 (0)	14.17 (18)	0.00 (0)	16.54 (21)	3.15 (4)	9.45 (12)	3.15 (4)	7.87 (10)	7.09 (9)	5.51 (7)	1.57 (2)	0.00 (0)	0.00 (0)	0.79 (1)	0.00 (0)	1.57 (2)	0.00 (0)	0.79 (1)	2.36 (3)
Coelomata																						
Deuterostomia																						
Chordata (48)	8.33 (4)	8.33 (4)	4.17 (2)	10.42 (5)	4.17 (2)	8.33 (4)	8.33 (4)	6.25 (3)	0.00 (0)	6.25 (3)	8.33 (4)	4.17. (2)	4.17 (2)	10.42 (5)	4.17 (2)	2.08 (1)	0.00 (0)	6.25 (3)	2.08 (1)	4.17 (2)	6.25 (3)	4.17 (2)
Echinodermata (2)	0.00 (0)	0.00 (0)	0.00 (0)	0.00 (0)	0.00 (0)	100.00 (2)	0.00 (0)	0.00 (0)	0.00 (0)	0.00 (0)	0.00 (0)	0.00 (0)	0.00 (0)	0.00 (0)	0.00 (0)	0.00 (0)	0.00 (0)	0.00 (0)	0.00 (0)	0.00 (0)	0.00 (0)	0.00 (0)
Hemichordata (3)	0.00 (0)	0.00 (0)	0.00 (0)	0.00 (0)	0.00 (0)	100.00 (3)	0.00 (0)	0.00 (0)	0.00 (0)	0.00 (0)	0.00 (0)	0.00 (0)	0.00 (0)	0.00 (0)	0.00 (0)	0.00 (0)	0.00 (0)	0.00 (0)	0.00 (0)	0.00 (0)	0.00 (0)	0.00 (0)
Protostomia																						
Annelida (55)	1.82 (1)	5.45 (3)	1.82 (1)	0.00 (0)	1.82 (1)	0.00 (0)	1.82 (1)	0.00 (0)	1.82 (1)	0.00 (0)	1.82 (1)	1.82 (1)	1.82 (1)	3.64 (2)	1.82 (1)	0.00 (0)	1.82 (1)	9.09 (5)	1.82 (1)	9.09 (5)	0.00 (0)	0.00 (0)
Echiura (1)	0.00 (0)	0.00 (0)	0.00 (0)	0.00 (0)	0.00 (0)	0.00 (0)	0.00 (0)	0.00 (0)	0.00 (0)	0.00 (0)	100.00 (1)	0.00 (0)	0.00 (0)	0.00 (0)	0.00 (0)	0.00 (0)	0.00 (0)	0.00 (0)	0.00 (0)	0.00 (0)	0.00 (0)	0.00 (0)
Brachiopoda (4)	0.00 (0)	0.00 (0)	0.00 (0)	0.00 (0)	0.00 (0)	0.00 (0)	0.00 (0)	0.00 (0)	0.00 (0)	0.00 (0)	0.00 (0)	0.00 (0)	0.00 (0)	0.00 (0)	0.00 (0)	0.00 (0)	0.00 (0)	0.00 (0)	25.00 (1)	0.00 (0)	0.00 (0)	0.00 (0)
Bryozoa (14)	7.14 (1)	14.29 (2)	0.00 (0)	7.14 (1)	0.00 (0)	7.14 (1)	14.29 (2)	7.14 (1)	21.43 (3)	14.29 (2)	14.29 (2)	0.00 (0)	14.29 (2)	0.00 (0)	0.00 (0)	0.00 (0)	0.00 (0)	0.00 (0)	0.00 (0)	0.00 (0)	7.14 (1)	0.00 (0)
Entoprocta (1)	0.00 (0)	0.00 (0)	0.00 (0)	0.00 (0)	0.00 (0)	0.00 (0)	0.00 (0)	0.00 (0)	0.00 (0)	0.00 (0)	0.00 (0)	0.00 (0)	0.00 (0)	0.00 (0)	0.00 (0)	0.00 (0)	0.00 (0)	0.00 (0)	0.00 (0)	0.00 (0)	0.00 (0)	0.00 (0)
Mollusca (102)	0.00 (0)	0.98 (1)	1.96 (2)	0.00 (0)	0.00 (0)	4.90 (5)	0.98 (1)	2.94 (3)	1.96 (2)	0.98 (1)	1.96 (2)	0.98 (1)	2.94 (3)	0.00 (0)	0.98 (1)	0.00 (0)	2.94 (3)	3.92 (4)	1.96 (2)	1.96 (2)	0.00 (0)	0.98 (1)
Myzostomida (3)	0.00 (0)	0.00 (0)	0.00 (0)	0.00 (0)	0.00 (0)	0.00 (0)	0.00 (0)	0.00 (0)	100.00 (3)	0.00 (0)	0.00 (0)	0.00 (0)	0.00 (0)	0.00 (0)	0.00 (0)	0.00 (0)	0.00 (0)	0.00 (0)	0.00 (0)	0.00 (0)	0.00 (0)	0.00 (0)
Nemertea (1)	0.00 (0)	0.00 (0)	0.00 (0)	0.00 (0)	0.00 (0)	0.00 (0)	0.00 (0)	0.00 (0)	0.00 (0)	0.00 (0)	0.00 (0)	0.00 (0)	0.00 (0)	0.00 (0)	0.00 (0)	0.00 (0)	0.00 (0)	0.00 (0)	0.00 (0)	0.00 (0)	0.00 (0)	0.00 (0)
Panarthropoda																						
Arthropoda (310)	4.19 (13)	4.19 (13)	5.48 (17)	1.61 (5)	0.65 (2)	6.13 (19)	5.16 (16)	0.65 (2)	3.23 (10)	3.55 (11)	6.13 (19)	4.19 (13)	3.23 (10)	2.26 (7)	5.48 (17)	1.61 (5)	6.77 (21)	1.94 (6)	13.87 (43)	4.84 (15)	1.94 (6)	1.94 (6)
Onychophora (1)	0.00 (0)	0.00 (0)	0.00 (0)	0.00 (0)	100.00 (1)	0.00 (0)	100.00 (1)	0.00 (0)	0.00 (0)	0.00 (0)	100.00 (1)	0.00 (0)	100.00 (1)	0.00 (0)	0.00 (0)	0.00 (0)	100.00 (1)	0.00 (0)	0.00 (0)	0.00 (0)	100.00 (1)	0.00 (0)
Tardigrada (1)	0.00 (0)	0.00 (0)	0.00 (0)	0.00 (0)	0.00 (0)	0.00 (0)	0.00 (0)	0.00 (0)	100.00 (1)	0.00 (0)	0.00 (0)	0.00 (0)	0.00 (0)	0.00 (0)	0.00 (0)	0.00 (0)	0.00 (0)	0.00 (0)	0.00 (0)	0.00 (0)	0.00 (0)	0.00 (0)
Priapulida (2)	0.00 (0)	0.00 (0)	0.00 (0)	0.00 (0)	0.00 (0)	0.00 (0)	0.00 (0)	0.00 (0)	0.00 (0)	0.00 (0)	0.00 (0)	0.00 (0)	0.00 (0)	0.00 (0)	0.00 (0)	0.00 (0)	0.00 (0)	0.00 (0)	0.00 (0)	0.00 (0)	0.00 (0)	0.00 (0)
Sipuncula (2)	0.00 (0)	0.00 (0)	0.00 (0)	0.00 (0)	0.00 (0)	0.00 (0)	0.00 (0)	0.00 (0)	0.00 (0)	0.00 (0)	0.00 (0)	0.00 (0)	0.00 (0)	0.00 (0)	0.00 (0)	0.00 (0)	0.00 (0)	0.00 (0)	0.00 (0)	0.00 (0)	0.00 (0)	0.00 (0)
Pseudocoelomata																						
Acanthocephala (25)	0.00 (0)	0.00 (0)	24.00 (6)	0.00 (0)	4.00 (1)	4.00 (1)	0.00 (0)	8.00 (2)	8.00 (2)	0.00 (0)	16.00 (4)	0.00 (0)	4.00 (1)	4.00 (1)	0.00 (0)	0.00 (0)	0.00 (0)	0.00 (0)	0.00 (0)	0.00 (0)	4.00 (1)	0.00 (0)
Cycliophora (1)	0.00 (0)	0.00 (0)	0.00 (0)	0.00 (0)	0.00 (0)	0.00 (0)	0.00 (0)	0.00 (0)	0.00 (0)	0.00 (0)	0.00 (0)	0.00 (0)	100.00 (1)	0.00 (0)	0.00 (0)	0.00 (0)	0.00 (0)	0.00 (0)	0.00 (0)	0.00 (0)	0.00 (0)	0.00 (0)
Kinorhyncha (1)	0.00 (0)	0.00 (0)	0.00 (0)	0.00 (0)	0.00 (0)	0.00 (0)	0.00 (0)	0.00 (0)	0.00 (0)	0.00 (0)	0.00 (0)	100.00 (1)	0.00 (0)	0.00 (0)	0.00 (0)	0.00 (0)	0.00 (0)	0.00 (0)	0.00 (0)	0.00 (0)	0.00 (0)	0.00 (0)
Nematoda (100)	3.00 (3)	12.00 (12)	3.00 (3)	3.00 (3)	2.00 (2)	2.00 (2)	11.00 (11)	14.00 (14)	6.00 (6)	2.00 (2)	2.00 (2)	6.00 (6)	0.00 (0)	1.00 (1)	1.00 (1)	1.00 (1)	2.00 (2)	1.00 (1)	1.00 (1)	0.00 (0)	5.00 (5)	1.00 (1)
Nematomorpha (1)	0.00 (0)	0.00 (0)	0.00 (0)	0.00 (0)	0.00 (0)	0.00 (0)	100.00 (1)	0.00 (0)	0.00 (0)	0.00 (0)	0.00 (0)	0.00 (0)	0.00 (0)	0.00 (0)	0.00 (0)	0.00 (0)	0.00 (0)	0.00 (0)	0.00 (0)	0.00 (0)	0.00 (0)	0.00 (0)
Rotifera (11)	0.00 (0)	0.00 (0)	0.00 (0)	0.00 (0)	0.00 (0)	0.00 (0)	9.09 (1)	0.00 (0)	9.09 (1)	0.00 (0)	0.00 (0)	9.09 (1)	0.00 (0)	9.09 (1)	0.00 (0)	0.00 (0)	0.00 (0)	0.00 (0)	0.00 (0)	0.00 (0)	0.00 (0)	0.00 (0)
Cnidaria (238)	3.78 (9)	1.26 (3)	7.56 (18)	2.16 (5)	6.30 (15)	1.26 (3)	8.82 (21)	6.72 (16)	2.94 (7)	0.84 (2)	1.68 (4)	1.68 (4)	7.14 (17)	0.84 (2)	2.10 (5)	1.26 (3)	1.26 (3)	1.26 (3)	0.84 (2)	1.68 (4)	5.46 (13)	1.26 (3)
Ctenophora (3)	0.00 (0)	0.00 (0)	0.00 (0)	0.00 (0)	0.00 (0)	0.00 (0)	0.00 (0)	0.00 (0)	0.00 (0)	0.00 (0)	33.33 (1)	0.00 (0)	0.00 (0)	0.00 (0)	0.00 (0)	0.00 (0)	0.00 (0)	0.00 (0)	0.00 (0)	0.00 (0)	0.00 (0)	0.00 (0)
Placozoa (4)	0.00 (0)	0.00 (0)	0.00 (0)	0.00 (0)	0.00 (0)	0.00 (0)	0.00 (0)	0.00 (0)	0.00 (0)	0.00 (0)	0.00 (0)	0.00 (0)	0.00 (0)	0.00 (0)	0.00 (0)	0.00 (0)	0.00 (0)	0.00 (0)	0.00 (0)	0.00 (0)	0.00 (0)	0.00 (0)
Porifera (11)	0.00 (0)	0.00 (0)	0.00 (0)	0.00 (0)	9.09 (1)	0.00 (0)	9.09 (1)	0.00 (0)	0.00 (0)	0.00 (0)	9.09 (1)	0.00 (0)	0.00 (0)	0.00 (0)	9.09 (1)	0.00 (0)	9.09 (1)	0.00 (0)	0.00 (0)	0.00 (0)	0.00 (0)	0.00 (0)

Comparisons were made for each phylum. One and two or more mismatches were estimated independently. The numbers in parentheses indicate the number of sequences that had the mismatches. The hierarchy of the NCBI taxonomy database is followed in this table.

A maximum of 16 fold-degeneracy was required to design the primers for the 18S ribosomal DNA (Table 1). For those phyla with ten or more sequences, at primer positions #1 and #5, all phyla had one or no mismatches for more than 90% of the sequences (Table 2). For primer position #2, this level of matching was exceeded in all of the phyla except the Chaetognatha (85% of sequences with one or no mismatches). For primer position #4, this level of matching was exceeded in all of the phyla except the Gastrotricha (71% of the sequences with one or no mismatches). For primer position #3, only the Chordata (89%) and the Gastrotricha (88%) had fewer than 90% of sequences with one or no mismatches.

A maximum of 48-fold degeneracy was required for designing primers for the 28S ribosomal DNA region (Table 1). For primer positions #3, #6, #8, #9, #10 and #11, all of the phyla had more than 90% of their sequences with one or no mismatches (Table 3). For four additional primer positions, this level of the matching was exceeded in all the phyla except the Chordata (90% for positions #2 and #7), Nematoda (86% for position #4), and Bryozoa (86% for position #5). For primer position #1, the only phyla with less than this level of matching were the Bryozoa (86%) and the Nematoda (88%).

### 2. Sliding window analyses of nucleotide diversity

**Figure 2:**
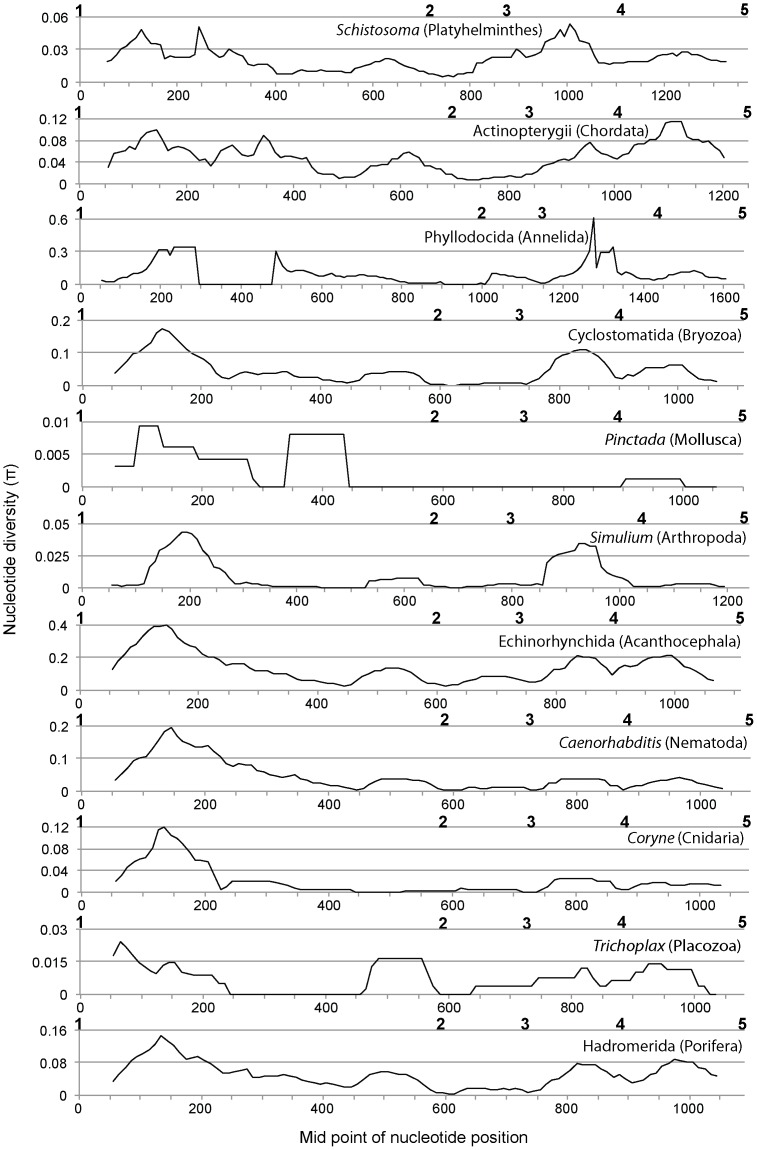
Sliding-window plots of nucleotide diversity (π) across the nuclear 18S ribosomal DNA of the 11 selected taxa. Positions of the newly designed primers are indicated above the X-axis in bold. Phylum of each taxonomic group is indicated in parentheses. The analyses were performed using sequences of 101 Schistosoma (Platyhelminthes), 257 Actinopterygii (Chordata), 214 Phyllodocida (Annelida), 20 Cyclostomatida (Bryozoa), 17 Pinctada (Mollusca), 27 Simulium (Arthropoda), 19 Echinorhynchida (Acanthocephala), 20 Caenorhabditis (Nematoda), 12 Coryne (Cnidaria), 9 Trichoplax (Placozoa), and 12 Hadromerida (Porifera).

**Figure 3:**
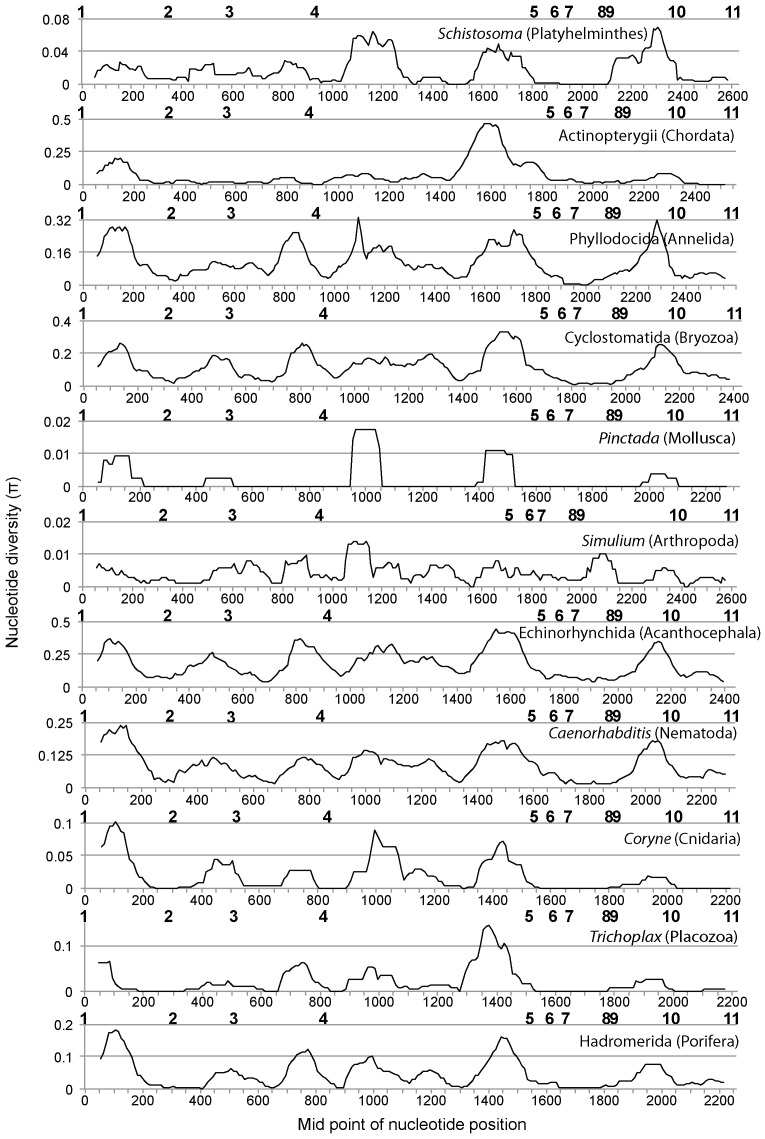
Sliding-window plots of nucleotide diversity (π) across the nuclear 28S ribosomal DNA of the 11 selected taxa. Positions of the newly designed primers are indicated above the X-axis in bold. Phylum of each taxonomic group is indicated in parentheses. The analyses were performed using sequences of 34 Schistosoma (Platyhelminthes), 4 Actinopterygii (Chordata), 16 Phyllodocida (Annelida), 6 Cyclostomatida (Bryozoa), 15 Pinctada (Mollusca), 21 Simulium (Arthropoda), 14 Echinorhynchida (Acanthocephala), 11 Caenorhabditis (Nematoda), 12 Coryne (Cnidaria), 4 Trichoplax (Placozoa), and 7 Hadromerida (Porifera).

Sliding window analyses of the 18S ribosomal DNA region showed a similar nucleotide diversity pattern for all the taxa analyzed in this study (Fig. 2). The highest nucleotide diversity was observed between nucleotide positions 50–250. The next peak was observed for the region between primers 3 and 4. More peaks were observed in the 28S ribosomal DNA region than in the 18S ribosomal DNA region, but again a similar nucleotide diversity pattern was observed across all the taxa (Fig. 3). A relatively high peak was observed for the region between primers 1 and 2, and subsequent peaks were observed for the region between primers 2 and 3 as well as between primers 3 and 4. For the region between primers 4 and 5, two to four peaks were observed. No peaks were observed for the region between primer positions 5 to 9. The last clear peak was observed for the region between primers 9 and 10. All of the primer regions were depicted on a secondary structure model of *Apis mellifera* ribosomal RNA genes derived from [22] (Figs. S1, S2, S3). This showed that the regions with higher nucleotide diversity were all around variable regions estimated from the secondary structure model (Figs. 1, 2, 3). All newly designed primers were located outside of these variable regions.

### 3. PCR and sequencing test

Several combinations of the primers were tested for 23 animals belonging to six phyla: Sipuncula (*Phascolosoma* sp.); Echinodermata [*Ophiocoma erinaceus* (brittlestar)]; Chordata [*Pseudamiops gracilicauda* (fish)]; Annelida [*Pherecardia striata* (Polychaeta), unidentified terebellid species (Polychaeta)]; Arthropoda [*Xanthias latifrons* (brachyuran crab), *Pilodius flavus* (brachyuran crab), *Liomera* sp. (brachyuran crab), *Carupa* sp. (brachyuran crab), unidentified pilumnid species (brachyuran crab), *Calcinus gouti* (anomuran crab), *Synalpheus* sp. (caridean shrimp), *Periclimenes* sp. (caridean shrimp), unidentified amphipod species]; Mollusca [*Cypraea helvola* (gastropod), *Cypraea fimbriata* (gastropod), *Trivia* sp. (gastropod), *Erato sandwichensis* (gastropod), unidentified haminoeid species (gastropod), *Berthellina* sp. (gastropod), *Chlamys* sp. (bivalve), *Lima* sp. (bivalve), unidentified lucinid species (bivalve)].

Based on these trials, we chose combinations of primers that run through a region with high nucleotide diversity (Figs. 2 and 3) and obtained PCR bands from all individuals using two primer combinations (18S #1 and 18S #2RC, 28S #8 and 28S #11RC), one for each gene. Although weak non-target bands were observed from a few individuals using the primer combinations of 18S #1 and 18S #2RC, clear electropherograms were obtained from most of the PCR products after being cut out from gels and sequenced. Clear single PCR products were also obtained using primer combinations of 28S #8 and 28S #11RC, and only *Pseudamiops gracilicauda* failed to yield clear electropherograms. These primers have less degeneracy, which reduces chances of co-amplification of non-target sequences. No amplification was observed from negative controls using any of the primer combinations.

Although we propose primer pairs 18S #1/#2RC and 28S #8/#11RC as good candidates for metazoan metagenetic analyses based on the likelihood that they will successfully amplify the target regions in most metazoans, some of the alternative primers in Table 1 might be more suitable in some cases. For example, two or more mismatches between the 18S primer (18S #2) sequences and their target regions were observed in Chaetognatha (Table 2). Because groups with higher mismatches will be less effectively amplified when the primers are used for metagenetic analysis [23], application of other primer combinations might sometimes be appropriate, depending on the community and taxa being analyzed. Another reason to use alternative primers is because of limitations of the sequencers. Commonly used second-generation sequencers, such as Roche 454, Illumina Solexa, and Applied Biosystems SOLiD, have length limitation not only for reading but also for library processing (length limitation for emulsion PCR and bridge amplification). Those limitations vary among the machines, although those limitations are getting smaller of late. From this standpoint, alternative primer combinations yielding shorter products might be better solutions depending on the machine being used. The approximate length of the amplicons can be estimated from Figures 1, 2, 3.

### 4. Comparison of suitability of different gene regions for metagenetic analyses

In the present study we have designed and tested the compatibility of primers for nuclear 18S and 28S ribosomal DNA sequences. However, other regions might be good targets for the metagenetic analyses depending on the goals of the study. We have listed five regions [mitochondrial COI, 12S, nuclear ITS (Internal Transcribed Spacer), 18S and 28S] as potential candidates and compared advantages and disadvantages of these regions as targets for metagenetic analyses (Table 4).

**Table 4:** Advantages and disadvantages of each gene region for metagenetic analyses.

	mtCOI	mt12S	ncITS	nc28S	nc18S
Evolutionary rate	Very fast	Fast	Slow	Slow	Very slow
Designing universal primers	Very difficult	Difficult	Easy	Easy	Very easy
Coding gene	Yes	Yes	No	Yes	Yes
Coding protein	Yes	No	No	No	No
Alignment	Easy	Very difficult	Very difficult	Easy	Easy

Closely related taxa are most reliably distinguished using regions with fast evolutionary rates. In this regard, the mitochondrial COI gene has an advantage over the other four genes [24], [25]. The nuclear 18S and 28S ribosomal genes have slower evolutionary rates [19] so that the ability to distinguish closely related taxa using these primers will generally be lower than for primers that target COI [25]. On the other hand, ease of designing universal primers is inversely related to evolutionary rate, and universality is important for metagenetic analyses of environmental samples. In this regard, the 18S and 28S genes have an advantage over COI.

The five regions also differ with respect to whether or not they are coding sequences. The nuclear ITS region is non-coding, making it difficult to identify informatically whether any sequence obtained is from the ITS region. Furthermore, because of the frequent occurrence of insertions and deletion in the nuclear ITS region, it is difficult to assign sequences to taxa by sequence similarity, especially to higher taxonomic levels. Nevertheless, the nuclear ITS region can potentially be a useful marker if a high-density of reference sequences are available for the target community and species.

Of the five regions, only the mitochondrial COI region is a protein-coding gene, which results in different rates of evolution for the different codon positions and thus the potential for information to be obtained for a wide range of taxonomic levels. For example, second codon information can be used to make assignments at higher taxonomic levels, whereas third codon information will be good for population or species level estimation [24].

Alignment of sequences is the first analytic step after sequence data are available. However, it is not always an easy procedure because of occurrence of indels. In this regard, the mitochondrial COI region has an advantage over the other regions because insertions and deletions are relatively uncommon in protein-coding gene sequences. In contrast, lengthy and numerous insertions and deletions can be expected within the mitochondrial 12S and nuclear ITS regions, making it difficult to get good alignments, especially across a large taxonomic span such as all metazoans.

Another criterion in the choice of gene region for metagenetics is the availability of reference sequence data. If a goal of a study is to assign sequences to taxonomic groupings, then the density of the reference sequence data will be important. In this regards, mitochondrial COI and nuclear 18S and 28S regions have advantages because of the availability of datasets [26], [27].

## Materials and Methods

### Designing PCR primers for the nuclear 18S and 28S ribosomal DNA

Two datasets containing nuclear 18S and 28S ribosomal DNA sequences (SSURef_106_full_align_tax_silva_trunc.fasta and LSURef_106_full_align_tax_silva_trunc.fasta) were downloaded from the SILVA database (www.arb-silva.de) [26] and metazoan sequences were extracted. No new sequences were generated in this study. Sequences without taxonomic information, such as those determined from environmental DNA, were removed. Sequences with ambiguities and some positions where all characters were “-” (gaps) were also removed using Mothur command screen.seqs and filter.seqs [28]. These filtered sequences were then imported to Geneious (Biomatters Ltd), and conserved regions suitable for designing primers were identified. In total, five and eight primer sites were found in the 18S and 28S ribosomal DNA regions, respectively (Table 1). Next, SILVA sequences that did not extend to those primer sites (short sequences) were removed from the dataset by using the Mothur command screen.seqs. We did not extend the primer search beyond the identified primer regions to keep the number of SILVA sequences as large as possible. As a result, we retained 14,503 18S and 1,073 28S metazoan nuclear ribosomal DNA sequences. Because of the limitations of the SILVA database, some regions of the 3′ and 5′ ends of genes were not included the analyses.

The newly identified primers in the prepared SILVA metazoan datasets were searched for each phylum using three criteria: no mismatches, one mismatch, and two or more mismatches. If >20% of the sequences of each phylum showed ≥1 mismatches, all the sequences of the phylum were extracted, and the sites with mismatches were inspected. If the mismatch at a single site was shared by >20% of the sequences of that phylum, the degeneracy of the primer was increased and the primer site search was repeated. This step was repeated until the prevalence of mismatches was <20% for all the phyla having >10 sequences. Some primer sites still had a mismatch rate of >20% after this procedure; in these cases, the indels or substitutions were shared by <20% of the sequences at multiple sites.

In addition to the developing these newly designed primers, we also performed this same compatibility test for previously reported universal primers [19]–[21] (Table S1, S2). After the first search, the degeneracy of the primers that had good compatibility was increased until the prevalence of the mismatches was <20% for the phyla having >10 sequences. Primers that anneal to regions outside of the prepared SILVA datasets were not considered. As a result, primer numbers 22, 24, 26 from [21] were retained as good candidates and numbered 28S #6, 28S #8, and 28S #10, respectively, in the present study. Primer number 28v [19] also had good compatibility with the SILVA datasets, but it was removed from the list because of degeneracy at both end positions of the primer. Other than these primers, good compatibility was not observed between previously published primers and the metazoans sequences we obtained from the SILVA datasets (Table S1, S2).

During preliminary analyses, we increased the degeneracy of the primers to decrease the mismatches by up to 10%. However, the highly degenerate primers failed to amplify individually extracted DNA because the degeneracy was too high. Therefore, we maintained a percentage of mismatches at a maximum of 20%.

### Sliding window analyses of nucleotide diversity

Sliding window analysis of nucleotide diversity was performed to determine a suitable region for metagenetic analyses. In total, 11 lower taxonomic groups, ranging from genus to class, were selected for this analysis (Figs. 2 and 3; the name of the taxon and number of sequences used for the analysis are listed in the figure legend). Although some of these taxa are terrestrial or parasitic, they were included because ancestors of these phyla were marine. First, sequences for these taxonomic groups were extracted from the datasets, and sequence alignment was performed using MAFFT E-INS-i [29]. Sequences outside the newly designed primer regions were removed from the datasets. Sliding window analysis of these datasets was performed using the *Drosophila* Polymorphism Database, SNP Graphics (http://dpdb.uab.es/dpdb/diversity.asp), with window length: 99 and step size: 10. During the analysis, we found some sequences with very large indels (sequence ID, 18S: AANH01015347.5743.7741, AANH01010553.44707.47729, and FJ196122.1.1563; 28S: AF154052.1.3517 and DQ790024.1.3760), which were removed from the datasets.

Compatibility test using PCR

The designed primers were tested for individuals belonging to various phyla. For the test, we chose the primer combinations that run through a region with high nucleotide diversity (18S: #1/#2RC; #3/#4RC, 28S: #1/#3RC; #2/#3RC; #2/#4RC; #3/#4RC; #4/#5RC; #4/#6RC; #4/#8RC; #5/#10RC; #6/#10RC; #7/#10RC; #7/#11RC; #8/#10RC; #8/#11RC; #9/#10RC; #9/#11RC). Extractions of DNA were performed using DNeasy Blood & Tissue Kit (Qiagen) following the manufacturer's protocol. PCR was done in a Veriti thermal cycler (Applied Biosystems), and reactions were carried out with a 15 µl reaction volume containing 9.8 µl of sterile, distilled H2O, 1.5 µl of 10×2 SA PCR buffer (Clontech), 1.2 µl of dNTP (2.5 mM each), 0.6 µl of each primer (5 µM), 0.3 µl of Advantage 2 DNA Polymerase Mix (Clontech), and 1.0 µl of the templates. A PCR mixture without template was also prepared as a negative control. Initial denaturation was carried out at 95°C for 10 min. The thermal-cycle profile for the PCR reaction was as follows (30 cycles): denaturation at 95°C for 10 s, annealing at 55°C for 30 s, and extension at 72°C for 60 s. PCR products were electrophoresed on a 1% TAE agarose gel together with Safe-Green (Applied Biological Materials Inc.) and visualized using a blue light transilluminator (Maestrogen: LB-16). Observed target bands were cut out from the gel and sent to Genomics BioSci & Tech (Taipei, Taiwan) for sequencing using standard Applied Biosystems protocols.

## Supporting Information

Figure S1 **Positions of the primers depicted on the secondary structure model of the 18S nuclear ribosomal RNA gene (domains I-III) of Apis mellifera (figure modified from** [**22**] **).**
(TIF)

Figure S2 **Positions of the primers depicted on the secondary structure model of the 28S nuclear ribosomal RNA gene (domains I-III) of Apis mellifera (figure modified from** [**22**] **).**
(TIF)

Figure S3 **Positions of the primers depicted on secondary structure model of the 28S nuclear ribosomal RNA gene (domains IV-VI) of Apis mellifera (figure modified from** [**22**] **).** Primers #8 and #9 overlap by 13 nt.
(TIF)

Table S1 Percentages of sequences, which showed mismatches between the previously reported primer [17] and target regions of the nuclear 18S ribosomal DNA sequences downloaded from the SILVA database. Comparisons were made for each phylum. One and two or more mismatches were estimated independently. The numbers in parentheses indicate the number of sequences that had the mismatches. The hierarchy of the NCBI taxonomy database is followed in this table.
(DOC)

Table S2 Percentages of sequences, which showed mismatches between the previously reported primer [17]–[19] and target region of the nuclear 28S ribosomal DNA sequences downloaded from the SILVA database. Comparisons were made for each phylum. One and two or more mismatches were estimated independently. The numbers in parentheses indicate the number of sequences that had the mismatches. The hierarchy of the NCBI taxonomy database is followed in this table.
(DOC)
