# Correction: PCR Primers for Metazoan Nuclear 18S and 28S Ribosomal DNA Sequences

**DOI:** 10.1371/journal.pone.0134314

**Published:** 2015-07-28

**Authors:** Ryuji J. Machida, Nancy Knowlton

The header “Forward primers (3' to 5')” should read “Forward primers (5' to 3'),” and the header “Reverse complement primers (3' to 5’)” should read “Reverse complement primers (5' to 3’).” The authors have provided a corrected version of Table 1 here.

**Table 1:** List of the 18S and 28S ribosomal DNA primers designed in this study. Primers 28S #6, 28S #8, and 28S #10 were modified from primers 22, 24, 26 **[21]**, respectively. Primers ultimately selected for best overall efficacy are highlighted in bold.

18S ribosomal DNA		
Forward primers (5' to 3')	Reverse complement primers (5' to 3')	Degeneracy
**#1: CTGGTGCCAGCAGCCGCGGYAA**		2
#2: AACTTAAAGRAATTGACGGA	**#2_RC: TCCGTCAATTYCTTTAAGTT**	2
#3: GYGGTGCATGGCCGTTSKTRGTT	#3_RC: AACYAMSAACGGCCATGCACCRC	16
#4: ATAACAGGTCWGTRATGCCCTYMG	#4_RC: CKRAGGGCATYACWGACCTGTTAT	16
	#5_RC: GTGTGYACAAAGGBCAGGGAC	6
28S ribosomal DNA		
Forward primers (5' to 3')	Reverse complement primers (5' to 3')	Degeneracy
#1: CCGTCTTGAAACACGGDCYRAG		6
#2: AGGGGCGAAAGACYAATCGAA	#2_RC: TTCGATTRGTCTTTCGCCCCT	2
#3: TTTTGGTAAGCAGAACTGGYG	#3_RC: CRCCAGTTCTGCTTACCAAAA	2
#4: GATCTYRGTGGYAGTAGCRAVT	#4_RC: ABTYGCTACTRCCACYRAGATC	48
#5: GGGAATCYRACTGTHTAATTAAA	#5_RC: TTTAATTADACAGTYRGATTCCC	12
#6: TGATTTCTGCCCAGTGCTYWGAAWGT	#6_RC: ACWTTCWRAGCACTGGGCAGAAATCA	8
#7: AACGGCGGRRGTAACTATGACTYT	#7_RC: ARAGTCATAGTTACYYCCGCCGTT	8
**#8: GGGAAAGAAGACCCTGTTGAG**	#8_RC: CTCAACAGGGTCTTCTTTCCC	1
#9: AAGACCCTGTTGAGYTTGACTCT	#9_RC: AGAGTCAARCTCAACAGGGTCTT	2
#10: GGGAGTTTGRCTGGGGCGG	#10_RC: CCGCCCCAGYCAAACTCCC	2
	**#11_RC: GCTTGGCBGCCACAAGCCAGTTA**	3
