# Supplementary figures and images for: PCR Primers for Metazoan Nuclear 18S and 28S Ribosomal DNA Sequences
Source: PLoS One. 2012 Sep 25;7(9):e46180. doi: 10.1371/journal.pone.0046180 (PMC3458000; doi:10.1371/journal.pone.0046180)

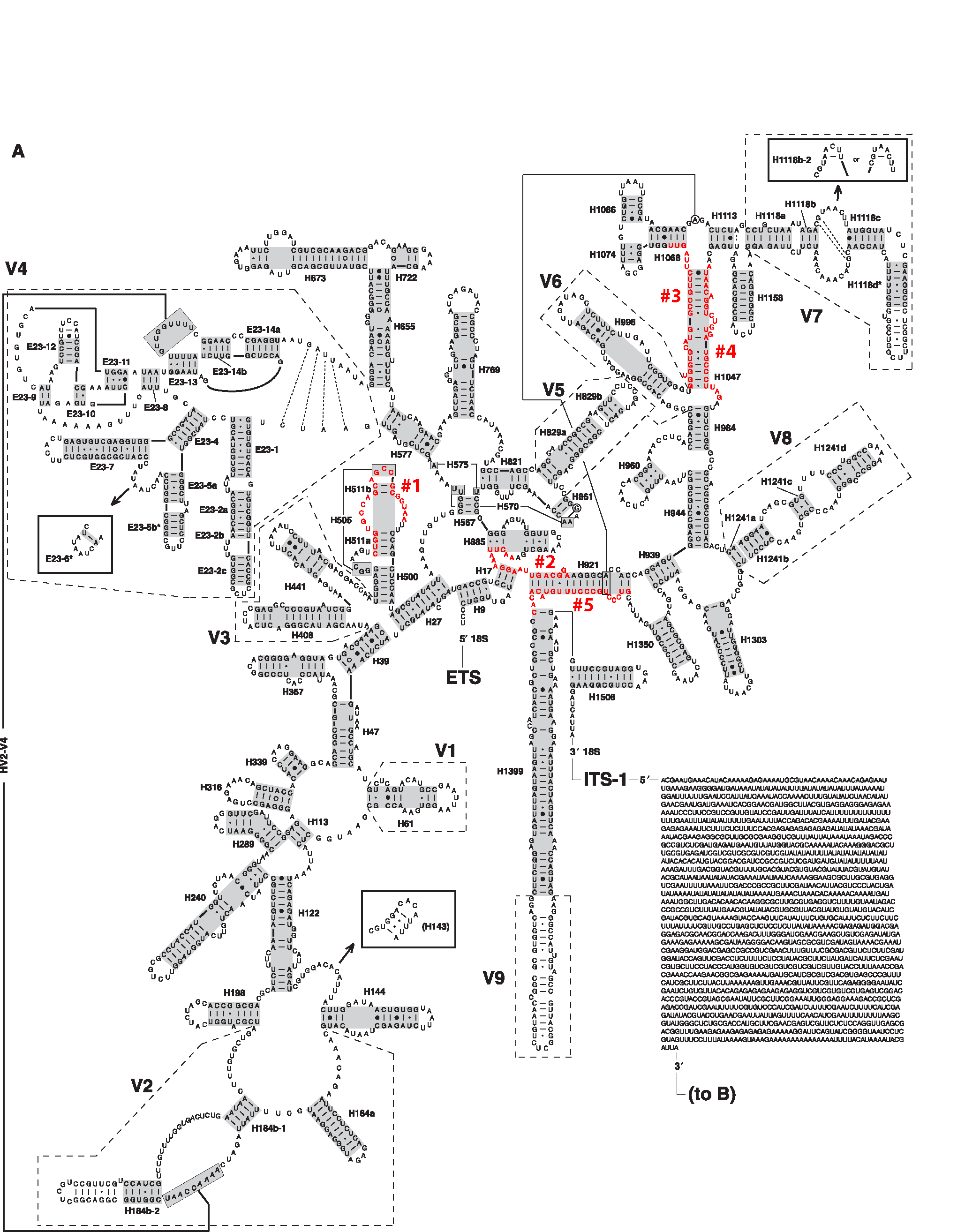

Supplement: Figure S1 — Positions of the primers depicted on the secondary structure model of the 18S nuclear ribosomal RNA gene (domains I-III) of Apis mellifera (figure modified from [22] ). (TIF) [file pone.0046180.s001.tif]

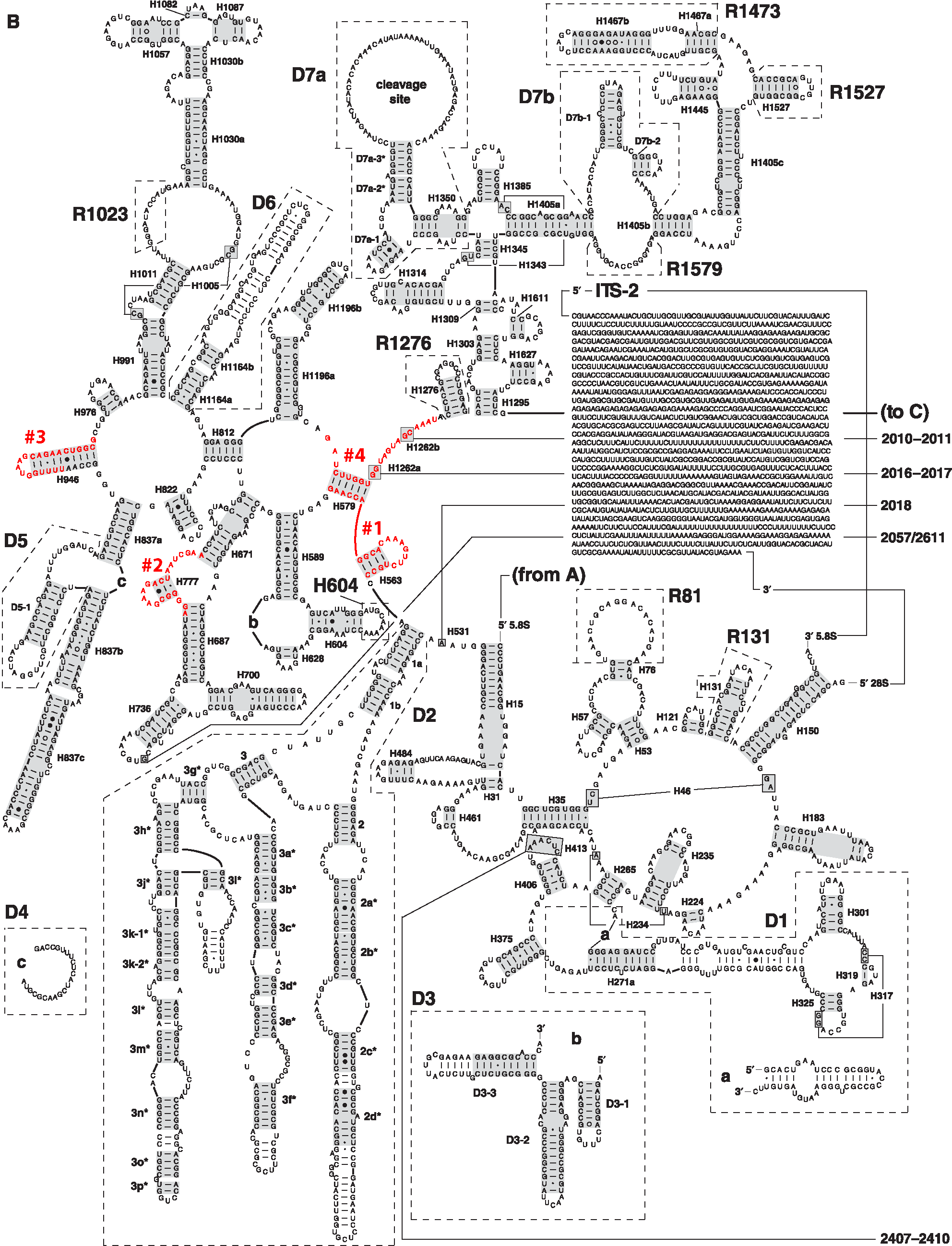

Supplement: Figure S2 — Positions of the primers depicted on the secondary structure model of the 28S nuclear ribosomal RNA gene (domains I-III) of Apis mellifera (figure modified from [22] ). (TIF) [file pone.0046180.s002.tif]

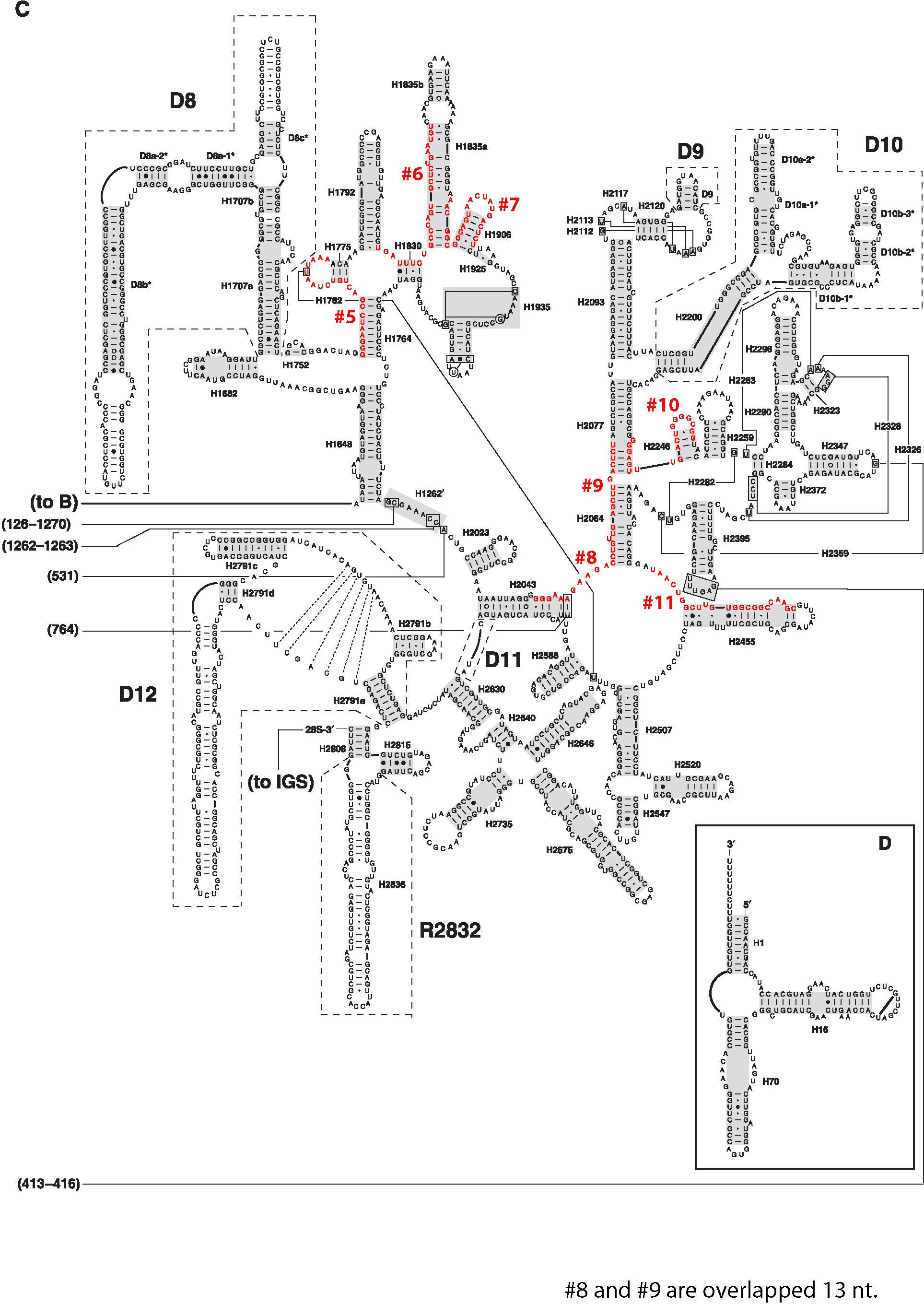

Supplement: Figure S3 — Positions of the primers depicted on secondary structure model of the 28S nuclear ribosomal RNA gene (domains IV-VI) of Apis mellifera (figure modified from [22] ). Primers #8 and #9 overlap by 13 nt. (TIF) [file pone.0046180.s003.tif]
