# Supplementary material for: PCR Primers for Metazoan Nuclear 18S and 28S Ribosomal DNA Sequences
Source: PLoS One. 2012 Sep 25;7(9):e46180. doi: 10.1371/journal.pone.0046180 (PMC3458000; doi:10.1371/journal.pone.0046180)
Supplement: Table S1 — Percentages of sequences, which showed mismatches between the previously reported primer [17] and target regions of the nuclear 18S ribosomal DNA sequences downloaded from the SILVA database. Comparisons were made for each phylum. One and two or more mismatches were estimated independently. The numbers in parentheses indicate the number of sequences that had the mismatches. The hierarchy of the NCBI taxonomy database is followed in this table. (DOC) [file pone.0046180.s004.doc]

***Table S1.* *Percentages of sequences, which showed mismatches between the previously reported primer [17] and target regions of the nuclear 18S ribosomal DNA sequences downloaded from the SILVA database.*** *Comparisons were made for each phylum. One and two or more mismatches were estimated independently. The numbers in parentheses indicate the number of sequences that had the mismatches. The hierarchy of the NCBI taxonomy database is followed in this table.*

Phylum (# sequences)

18b 18j (18k) 18O-C 18Q-C

% (# Sequences) % (# Sequences) % (# Sequences) % (# Sequences)

One Two or more One Two or more One Two or more One Two or more

Total

Metazoa

Eumetazoa

Bilateria (18) 0.00 (0) 100.00 (18) 27.78 (5) 0.00 (0) 11.11 (2) 50.00 (9) 38.89 (7) 5.56 (1)

Acoelomata

Platyhelminthes (1003) 0.00 (0) 100.00 (1003) 29.91 (300) 18.64 (187) 62.31 (625) 32.70 (328) 3.99 (40) 1.99 (20)

Coelomata

Deuterostomia

Chaetognatha (20) 0.00 (0) 100.00 (20) 5.00 (1) 20.00 (4) 95.00 (19) 5.00 (1) 0.00 (0) 5.00 (1)

Chordata (763) 32.90 (251) 25.82 (197) 5.37 (41) 10.48 (80) 48.75 (372) 8.78 (67) 11.80 (90) 6.82 (52)

Echinodermata (156) 0.00 (0) 100.00 (156) 3.85 (6) 1.92 (3) 3.85 (6) 14.10 (22) 0.64 (1) 0.00 (0)

Hemichordata (23) 0.00 (0) 100.00 (23) 0.00 (0) 4.35 (1) 13.04 (3) 0.00 (0) 0.00 (0) 34.78 (8)

Xenoturbellida (1) 0.00 (0) 100.00 (1) 0.00 (0) 0.00 (0) 0.00 (0) 0.00 (0) 0.00 (0) 0.00 (0)

Protostomia

Annelida (983) 0.00 (0) 99.90 (982) 4.07 (40) 3.76 (37) 34.59 (340) 9.56 (94) 1.83 (18) 1.12 (11)

Echiura (4) 0.00 (0) 100.00 (4) 0.00 (0) 0.00 (0) 100.00 (4) 0.00 (0) 0.00 (0) 0.00 (0)

Brachiopoda (41) 2.44 (1) 97.56 (40) 7.32 (3) 0.00 (0) 2.44 (1) 0.00 (0) 2.44 (1) 2.44 (1)

Bryozoa (47) 0.00 (0) 100.00 (47) 2.13 (1) 4.26 (2) 0.00 (0) 91.49 (43) 12.77 (6) 2.13 (1)

Entoprocta (14) 0.00 (0) 100.00 (14) 0.00 (0) 0.00 (0) 7.14 (1) 0.00 (0) 0.00 (0) 0.00 (0)

Mollusca (887) 0.00 (0) 100.00 (887) 23.68 (210) 6.88 (61) 17.47 (155) 9.70 (86) 5.98 (53) 1.01 (9)

Myzostomida (36) 0.00 (0) 100.00 (36) 100.00 (36) 0.00 (0) 19.44 (7) 80.56 (29) 100.00 (36) 0.00 (0)

Nemertea (34) 0.00 (0) 100.00 (34) 0.00 (0) 11.76 (4) 2.94 (1) 2.94 (1) 0.00 (0) 0.00 (0)

Panarthropoda

Arthropoda (7591) 1.50 (114) 98.49 (7476) 7.35 (558) 2.45 (186) 22.16 (1682) 24.34 (1848) 11.84 (899) 2.92 (222)

Onychophora (2) 0.00 (0) 100.00 (2) 0.00 (0) 0.00 (0) 　　0.00 (0) 100.00 (2) 0.00 (0) 0.00 (0)

Tardigrada (102) 0.00 (0) 100.00 (102) 1.96 (2) 0.00 (0) 31.37 (32) 18.63 (19) 1.96 (2) 0.00 (0)

Priapulida (6) 0.00 (0) 100.00 (6) 0.00 (0) 0.00 (0) 0.00 (0) 16.67 (1) 0.00 (0) 0.00 (0)

Sipuncula (16) 0.00 (0) 100.00 (16) 0.00 (0) 0.00 (0) 31.25 (5) 0.00 (0) 0.00 (0) 0.00 (0)

Pseudocoelomata

Acanthocephala (46) 0.00 (0) 100.00 (46) 0.00 (0) 100.00 (46) 10.87 (5) 89.13 (41) 89.13 (41) 8.70 (4)

Cycliophora (18) 0.00 (0) 100.00 (18) 100.00 (18) 0.00 (0) 0.00 (0) 　100.00 (18) 100.00 (18) 0.00 (0)

Gastrotricha (17) 0.00 (0) 100.00 (17) 0.00 (0) 5.88 (1) 41.18 (7) 11.76 (2) 35.29 (6) 5.88 (1)

Kinorhyncha (6) 0.00 (0) 100.00 (6) 0.00 (0) 0.00 (0) 0.00 (0) 　100.00 (6) 0.00 (0) 0.00 (0)

Loricifera (1) 0.00 (0) 100.00 (1) 0.00 (0) 0.00 (0) 100.00 (1) 0.00 (0) 100.00 (1) 0.00 (0)

Micrognathozoa (2) 0.00 (0) 100.00 (2) 0.00 (0) 0.00 (0) 50.00 (1) 50.00 (1) 0.00 (0) 0.00 (0)

Nematoda (1375) 15.13 (208) 84.00 (1155) 24.22 (333) 6.84 (94) 16.00 (220) 67.64 (930) 56.44 (776) 5.89 (81)

Nematomorpha (11) 0.00 (0) 100.00 (11) 0.00 (0) 9.09 (1) 0.00 (0) 0.00 (0) 18.18 (2) 0.00 (0)

Rotifera (68) 0.00 (0) 100.00 (68) 2.94 (2) 11.76 (8) 88.24 (60) 11.76 (8) 0.00 (0) 8.82 (6)

Cnidaria (969) 0.00 (0) 100.00 (969) 9.70 (94) 8.77 (85) 61.09 (592) 34.98 (339) 67.60 (655) 24.77 (240)

Ctenophora (18) 0.00 (0) 100.00 (18) 5.56 (1) 94.44 (17) 0.00 (0) 　100.00 (18) 100.00 (18) 0.00 (0)

Mesozoa (5) 0.00 (0) 100.00 (5) 0.00 (0) 100.00 (5) 20.00 (1) 80.00 (4) 40.00 (2) 0.00 (0)

Placozoa (9) 0.00 (0) 100.00 (9) 0.00 (0) 0.00 (0) 0.00 (0) 　100.00 (9) 100.00 (9) 0.00 (0)

Porifera (211) 0.00 (0) 100.00 (211) 3.32 (7) 7.58 (16) 75.36 (159) 19.43 (41) 83.89 (177) 10.90 (23)

–––––––––––––––––––––––––––––––––––––––––––––––––––––––––––––––––––––––––––––––––––––––––––––––––––––––––––––––––––––––––––––––––––––––––––––––––
