# Supplementary material for: PCR Primers for Metazoan Nuclear 18S and 28S Ribosomal DNA Sequences
Source: PLoS One. 2012 Sep 25;7(9):e46180. doi: 10.1371/journal.pone.0046180 (PMC3458000; doi:10.1371/journal.pone.0046180)
Supplement: Table S2 — Percentages of sequences, which showed mismatches between the previously reported primer [17]–[19] and target region of the nuclear 28S ribosomal DNA sequences downloaded from the SILVA database. Comparisons were made for each phylum. One and two or more mismatches were estimated independently. The numbers in parentheses indicate the number of sequences that had the mismatches. The hierarchy of the NCBI taxonomy database is followed in this table. (DOC) [file pone.0046180.s005.doc]

***Table S2. Percentages of sequences, which showed mismatches between the previously reported primer [17]-[19] and target region of the nuclear 28S ribosomal DNA sequences downloaded from the SILVA database.*** *Comparisons were made for each phylum. One and two or more mismatches were estimated independently. The numbers in parentheses indicate the number of sequences that had the mismatches. The hierarchy of the NCBI taxonomy database is followed in this table.*

––––––––––––––––––––––––––––––––––––––––––––––––––––––––––––––––––––––––––––––––––––––––––––––––––––––––––––––––––––––––––––––––––––––––––––––––––––––––––––––––––––––––––––––––––––––––––––––––––––––––––––––––––––––––––––––––––––––––––––––––––––

Phylum (# sequences)

28v (gg)* 28w (hh)* 28z (dd)* 28ee (ff)* 28ll (mm)* 10 ^#^ 12 ^#^ 14 ^#^

% (# Sequences) % (# Sequences) % (# Sequences) % (# Sequences) % (# Sequences) % (# Sequences) % (# Sequences) % (# Sequences)

One Two or more One Two or more One Two or more One Two or more One Two or more One Two or more One Two or more One Two or more

––––––––––––––––––––––––––––––––––––––––––––––––––––––––––––––––––––––––––––––––––––––––––––––––––––––––––––––––––––––––––––––––––––––––––––––––––––––––––––––––––––––––––––––––––––––––––––––––––––––––––––––––––––––––––––––––––––––––––––––––––––

Total

Metazoa

Eumetazoa

Acoelomata

Platyhelminthes (127) 5.51 (7) 3.15 (4) 82.68(105) 1.57(2) 77.17 (98) 7.87 (10) 53.54 (68) 0.79 (1) 53.54 (68) 46.46 (59) 2.36 (3) 2.36 (3) 7.09 (9) 0.79 (1) 53.54 (68) 0.79 (1)

Coelomata

Deuterostomia

Chordata (48) 4.17 (2) 6.25 (3) 10.42 (5) 4.17 (2) 20.83 (10) 10.42 (5) 20.83 (10) 10.42 (5) 12.50 (6) 12.50 (6) 25.00 (12) 8.33 (4) 2.08 (1) 10.42 (5) 16.67 (8) 8.33 (4)

Echinodermata (2) 0.00 (0) 0.00 (0) 0.00 (0) 0.00 (0) 50.00 (1) 0.00 (0) 0.00 (0) 0.00 (0) 100.00 (2) 0.00 (0) 50.00 (1) 0.00 (0) 0.00 (0) 0.00 (0) 0.00 (0) 0.00 (0)

Hemichordata (3) 0.00 (0) 0.00 (0) 100.00 (3) 0.00 (0) 0.00 (0) 0.00 (0) 0.00 (0) 0.00 (0) 66.67 (2) 0.00 (0) 0.00 (0) 0.00 (0) 0.00 (0) 0.00 (0) 0.00 (0) 0.00 (0)

Protostomia

Annelida (55) 5.45 (3) 1.82 (1) 63.64 (35) 21.82 (12) 14.55 (8) 7.27 (4) 12.73 (7) 14.55 (8) 74.55 (41) 23.64 (13) 10.91 (6) 5.45 (3) 20.00 (11) 0.00 (0) 12.73 (7) 14.55 (8)

Echiura (1) 0.00 (0) 0.00 (0) 0.00 (0) 0.00 (0) 0.00 (0) 0.00 (0) 0.00 (0) 0.00 (0) 100.00 (1) 0.00 (0) 0.00 (0) 0.00 (0) 100.00 (1) 0.00 (0) 0.00 (0) 0.00 (0)

Brachiopoda (4) 0.00 (0) 0.00 (0) 100.00 (4) 0.00 (0) 0.00 (0) 0.00 (0) 75.00 (3) 0.00 (0) 75.00 (3) 25.00 (1) 0.00 (0) 0.00 (0) 25.00 (1) 0.00 (0) 75.00 (3) 0.00 (0)

Bryozoa (14) 14.29 (2) 0.00 (0) 100.00 (14) 0.00 (0) 7.14 (1) 14.29 (2) 35.71 (5) 21.43 (3) 71.43 (10) 21.43 (3) 14.29 (2) 14.29 (2) 0.00 (0) 7.14 (1) 35.71 (5) 7.14 (1)

Entoprocta (1) 0.00 (0) 0.00 (0) 100.00 (1) 0.00 (0) 0.00 (0) 0.00 (0) 0.00 (0) 0.00 (0) 100.00 (1) 0.00 (0) 0.00 (0) 0.00 (0) 100.00 (1) 0.00 (0) 0.00 (0) 0.00 (0)

Mollusca (102) 1.96 (2) 0.00 (0) 77.45 (79) 9.80 (10) 1.96 (2) 5.88 (6) 30.39 (31) 0.98 (1) 90.20 (92) 7.84 (8) 4.90 (5) 0.98 (1) 23.53 (24) 1.96 (2) 28.43 (29) 0.98 (1)

Myzostomida (3) 0.00 (0) 0.00 (0) 0.00 (0) 0.00 (0) 0.00 (0) 0.00 (0) 0.00 (0) 0.00 (0) 0.00 (0) 100.00 (3) 0.00 (0) 0.00 (0) 0.00 (0) 0.00 (0) 0.00 (0) 0.00 (0)

Nemertea (1) 0.00 (0) 0.00 (0) 100.00 (1) 0.00 (0) 0.00 (0) 0.00 (0) 0.00 (0) 0.00 (0) 100.00 (1) 0.00 (0) 0.00 (0) 0.00 (0) 0.00 (0) 0.00 (0) 0.00 (0) 0.00 (0)

Panarthropoda

Arthropoda (310) 1.61 (5) 4.84 (15) 27.10 (84) 15.81 (49) 25.48 (79) 7.74 (24) 14.84 (46) 9.03 (28) 30.97 (96) 9.35 (29) 24.84 (77) 5.81 (18) 4.52 (14) 0.97 (3) 14.84 (46) 9.03 (28)

Onychophora (1) 0.00 (0) 0.00 (0) 0.00 (0) 100.00 (1) 100.00 (1) 0.00 (0) 0.00 (0) 0.00 (0) 0.00 (0) 100.00 (1) 100.00 (1) 0.00 (0) 0.00 (0) 0.00 (0) 0.00 (0) 0.00 (0)

Tardigrada (1) 0.00 (0) 0.00 (0) 0.00 (0) 0.00 (0) 0.00 (0) 100.00 (1) 0.00 (0) 0.00 (0) 0.00 (0) 100.00 (1) 0.00 (0) 0.00 (0) 0.00 (0) 0.00 (0) 0.00 (0) 0.00 (0)

Priapulida (2) 0.00 (0) 0.00 (0) 100.00 (2) 0.00 (0) 0.00 (0) 0.00 (0) 0.00 (0) 0.00 (0) 100.00 (2) 0.00 (0) 0.00 (0) 0.00 (0) 0.00 (0) 0.00 (0) 0.00 (0) 0.00 (0)

Sipuncula (2) 0.00 (0) 0.00 (0) 50.00 (1) 0.00 (0) 0.00 (0) 50.00 (1) 0.00 (0) 0.00 (0) 100.00 (2) 0.00 (0) 0.00 (0) 0.00 (0) 0.00 (0) 0.00 (0) 0.00 (0) 0.00 (0)

Pseudocoelomata

Acanthocephala (25) 76.00 (19) 16.00 (4) 100.00 (25) 0.00 (0) 0.00 (0) 100.00 (25) 32.00 (8) 40.00 (10) 4.00 (1) 96.00 (24) 76.00 (19) 20.00 (5) 52.00 (13) 48.00 (12) 32.00 (8) 40.00 (10)

Cycliophora (1) 0.00 (0) 0.00 (0) 0.00 (0) 100.00 (1) 100.00 (1) 0.00 (0) 0.00 (0) 0.00 (0) 100.00 (1) 0.00 (0) 100.00 (1) 0.00 (0) 0.00 (0) 0.00 (0) 0.00 (0) 0.00 (0)

Kinorhyncha (1) 0.00 (0) 0.00 (0) 100.00 (1) 0.00 (0) 100.00 (1) 0.00 (0) 100.00 (1) 0.00 (0) 0.00 (0) 100.00 (1) 0.00 (0) 0.00 (0) 0.00 (0) 0.00 (0) 100.00 (1) 0.00 (0)

Nematoda (100) 41.00 (41) 29.00 (29) 60.00 (60) 12.00 (12) 39.00 (39) 48.00 (48) 56.00 (56) 7.00 (7) 62.00 (62) 36.00 (36) 40.00 (40) 16.00 (16) 51.00 (51) 34.00 (34) 55.00 (55) 7.00 (7)

Nematomorpha (1) 100.00 (1) 0.00 (0) 0.00 (0) 100.00 (1) 0.00 (0) 0.00 (0) 0.00 (0) 0.00 (0) 0.00 (0) 100.00 (1) 0.00 (0) 0.00 (0) 0.00 (0) 0.00 (0) 0.00 (0) 0.00 (0)

Rotifera (11) 0.00 (0) 54.55 (6) 54.55 (6) 0.00 (0) 9.09 (1) 45.45 (5) 45.45 (5) 0.00 (0) 54.55 (6) 45.45 (5) 0.00 (0) 45.45 (5) 9.09 (1) 0.00 (0) 45.45 (5) 0.00 (0)

Cnidaria (238) 10.50 (25) 2.10 (5) 10.92 (26) 2.52 (6) 10.50 (25) 6.30 (15) 65.97 (157) 27.31 (65) 89.50 (213) 10.50 (25) 5.04 (12) 1.26 (3) 73.11 (174) 11.76 (28) 27.73 (66) 6.72 (16)

Ctenophora (3) 0.00 (0) 0.00 (0) 0.00 (0) 0.00 (0) 0.00 (0) 0.00 (0) 0.00 (0) 0.00 (0) 100.00 (3) 0.00 (0) 0.00 (0) 0.00 (0) 33.33 (1) 0.00 (0) 0.00 (0) 0.00 (0)

Placozoa (4) 0.00 (0) 25.00 (1) 100.00 (4) 0.00 (0) 0.00 (0) 0.00 (0) 0.00 (0) 0.00 (0) 0.00 (0) 100.00 (4) 0.00 (0) 0.00 (0) 100.00 (4) 0.00 (0) 0.00 (0) 0.00 (0)

Porifera (11) 0.00 (0) 0.00 (0) 72.73 (8) 18.18 (2) 9.09 (1) 81.82 (9) 9.09 (1) 9.09 (1) 27.27 (3) 72.73 (8) 18.18 (2) 0.00 (0) 27.27 (3) 0.00 (0) 9.09 (1) 9.09 (1)

––––––––––––––––––––––––––––––––––––––––––––––––––––––––––––––––––––––––––––––––––––––––––––––––––––––––––––––––––––––––––––––––––––––––––––––––––––––––––––––––––––––––––––––––––––––––––––––––––––––––––––––––––––––––––––––––––––––––––––––––––––

* Hillis and Dixon 1991

^#^ Van der Auwera 1994

^†^ Philippe et al. 1994

***Table S2. Continued***

––––––––––––––––––––––––––––––––––––––––––––––––––––––––––––––––––––––––––––––––––––––––––––––––––––––––––––––––––––––––––––––––––––––––––––––––––––––––––––––––––––––––

Phylum (# sequences)

16 ^#^ 18 ^#^ 20 ^#^ C8^†^ D8^†^

% (# Sequences) % (# Sequences) % (# Sequences) % (# Sequences) % (# Sequences)

One Two or more One Two or more One Two or more One Two or more One Two or more

––––––––––––––––––––––––––––––––––––––––––––––––––––––––––––––––––––––––––––––––––––––––––––––––––––––––––––––––––––––––––––––––––––––––––––––––––––––––––––––––––––––––

Total

Metazoa

Eumetazoa

Acoelomata

Platyhelminthes (127) 80.31 (102) 14.96 (19) 15.75 (20) 3.15 (4) 53.54 (68) 35.43 (45) 60.63 (77) 19.69 (25) 1.57 (2) 98.43 (125)

Coelomata

Deuterostomia

Chordata (48) 6.25 (3) 4.17 (2) 6.25 (3) 6.25 (3) 14.58 (7) 6.25 (3) 81.25 (39) 18.75 (9) 8.33 (4) 20.83 (10)

Echinodermata (2) 0.00 (0) 0.00 (0) 50.00 (1) 0.00 (0) 50.00 (1) 0.00 (0) 50.00 (1) 0.00 (0) 50.00 (1) 50.00 (1)

Hemichordata (3) 66.67 (2) 0.00 (0) 33.33 (1) 0.00 (0) 66.67 (2) 0.00 (0) 100.00 (3) 0.00 (0) 0.00 (0) 100.00 (3)

Protostomia

Annelida (55) 21.82 (12) 1.82 (1) 1.82 (1) 0.00 (0) 14.55 (8) 14.55 (8) 14.55 (8) 14.55 (8) 65.45 (36) 32.73 (18)

Echiura (1) 100.00 (1) 0.00 (0) 0.00 (0) 0.00 (0) 0.00 (0) 0.00 (0) 0.00 (0) 0.00 (0) 100.00 (1) 0.00 (0)

Brachiopoda (4) 25.00 (1) 0.00 (0) 0.00 (0) 0.00 (0) 25.00 (1) 0.00 (0) 25.00 (1) 0.00 (0) 100.00 (4) 0.00 (0)

Bryozoa (14) 0.00 (0) 28.57 (4) 7.14 (1) 7.14 (1) 21.43 (3) 0.00 (0) 21.43 (3) 7.14 (1) 21.43 (3) 78.57 (11)

Entoprocta (1) 0.00 (0) 0.00 (0) 0.00 (0) 0.00 (0) 100.00 (1) 0.00 (0) 100.00 (1) 0.00 (0) 100.00 (1) 0.00 (0)

Mollusca (102) 56.86 (58) 0.98 (1) 13.73 (14) 0.98 (1) 6.86 (7) 5.88 (6) 6.86 (7) 7.84 (8) 61.76 (63) 37.25 (38)

Myzostomida (3) 0.00 (0) 0.00 (0) 66.67 (2) 0.00 (0) 0.00 (0) 100.00 (3) 0.00 (0) 100.00 (3) 0.00 (0) 100.00 (3)

Nemertea (1) 0.00 (0) 0.00 (0) 0.00 (0) 0.00 (0) 0.00 (0) 0.00 (0) 0.00 (0) 0.00 (0) 0.00 (0) 100.00 (1)

Panarthropoda

Arthropoda (310) 67.10 (208) 18.71 (58) 40.00 (124) 2.58 (8) 21.61 (67) 9.03 (28) 78.71 (244) 11.94 (37) 0.00 (0) 100.00 (310)

Onychophora (1) 100.00 (1) 0.00 (0) 100.00 (1) 0.00 (0) 0.00 (0) 100.00 (1) 0.00 (0) 100.00 (1) 0.00 (0) 100.00 (1)

Tardigrada (1) 100.00 (1) 0.00 (0) 0.00 (0) 0.00 (0) 0.00 (0) 100.00 (1) 100.00 (1) 0.00 (0) 0.00 (0) 100.00 (1)

Priapulida (2) 0.00 (0) 0.00 (0) 0.00 (0) 0.00 (0) 0.00 (0) 0.00 (0) 0.00 (0) 0.00 (0) 100.00 (2) 0.00 (0)

Sipuncula (2) 50.00 (1) 0.00 (0) 0.00 (0) 0.00 (0) 100.00 (2) 0.00 (0) 100.00 (2) 0.00 (0) 50.00 (1) 50.00 (1)

Pseudocoelomata

Acanthocephala (25) 16.00 (4) 84.00 (21) 8.00 (2) 0.00 (0) 36.00 (9) 64.00 (16) 40.00 (10) 60.00 (15) 0.00 (0) 100.00 (25)

Cycliophora (1) 100.00 (1) 0.00 (0) 0.00 (0) 0.00 (0) 0.00 (0) 0.00 (0) 0.00 (0) 0.00 (0) 0.00 (0) 100.00 (1)

Kinorhyncha (1) 0.00 (0) 0.00 (0) 0.00 (0) 0.00 (0) 100.00 (1) 0.00 (0) 100.00 (1) 0.00 (0) 100.00 (1) 0.00 (0)

Nematoda (100) 14.00 (14) 5.00 (5) 19.00 (19) 10.00 (10) 26.00 (26) 10.00 (10) 64.00 (64) 12.00 (12) 0.00 (0) 100.00 (100)

Nematomorpha (1) 0.00 (0) 0.00 (0) 100.00 (1) 0.00 (0) 0.00 (0) 100.00 (1) 0.00 (0) 100.00 (1) 0.00 (0) 100.00 (1)

Rotifera (11) 54.55 (6) 0.00 (0) 9.09 (1) 0.00 (0) 9.09 (1) 45.45 (5) 9.09 (1) 45.45 (5) 0.00 (0) 100.00 (11)

Cnidaria (238) 13.87 (33) 2.52 (6) 45.38 (108) 2.94 (7) 7.98 (19) 2.52 (6) 7.56 (18) 4.20 (10) 0.00 (0) 100.00 (238)

Ctenophora (3) 0.00 (0) 0.00 (0) 0.00 (0) 0.00 (0) 0.00 (0) 0.00 (0) 0.00 (0) 0.00 (0) 0.00 (0) 100.00 (3)

Placozoa (4) 0.00 (0) 0.00 (0) 0.00 (0) 0.00 (0) 75.00 (3) 25.00 (1) 0.00 (0) 50.00 (2) 0.00 (0) 100.00 (4)

Porifera (11) 18.18 (2) 0.00 (0) 9.09 (1) 0.00 (0) 36.36 (4) 45.45 (5) 81.82 (9) 0.00 (0) 0.00 (0) 100.00 (11)

––––––––––––––––––––––––––––––––––––––––––––––––––––––––––––––––––––––––––––––––––––––––––––––––––––––––––––––––––––––––––––––––––––––––––––––––––––––––––––––––––––––––––––––––––––––––––––
